# WTD-PSD: Presentation of Novel Feature Extraction Method Based on Discrete Wavelet Transformation and Time-Dependent Power Spectrum Descriptors for Diagnosis of Alzheimer's Disease

**DOI:** 10.1155/2022/9554768

**Published:** 2022-05-11

**Authors:** Ali Taghavirashidizadeh, Fatemeh Sharifi, Seyed Amir Vahabi, Aslan Hejazi, Mehrnaz SaghabTorbati, Amin Salih Mohammed

**Affiliations:** ^1^Islamic Azad University, Central Tehran Branch (IAUCTB), Department of Electrical and Electronics Engineering, Tehran, Iran; ^2^Department of Electrical Engineering, University of Applied Science and Technology, Bushehr, Iran; ^3^Department of Computer Engineering, Deylaman Institute of Higher Education, Lahijan, Iran; ^4^Department of Electrical Engineering, Islamic Azad University, Science and Research Branch, Tehran, Iran; ^5^Department of Biomedical Engineering, Faculty of Electrical Engineering, Iran University of Science and Technology, Tehran, Iran; ^6^Department of Computer Engineering, College of Engineering and Computer Science, Lebanese French University, Erbil, Kurdistan Region, Iraq; ^7^Department of Software and Informatics Engineering, Salahaddin University, Erbil, Kurdistan Region, Iraq

## Abstract

Alzheimer's disease (AD) is a type of dementia that affects the elderly population. A machine learning (ML) system has been trained to recognize particular patterns to diagnose AD using an algorithm in an ML system. As a result, developing a feature extraction approach is critical for reducing calculation time. The input image in this article is a Two-Dimensional Discrete Wavelet (2D-DWT). The Time-Dependent Power Spectrum Descriptors (TD-PSD) model is used to represent the subbanded wavelet coefficients. The principal property vector is made up of the characteristics of the TD-PSD model. Based on classification algorithms, the collected characteristics are applied independently to present AD classifications. The categorization is used to determine the kind of tumor. The TD-PSD method was used to extract wavelet subbands features from three sets of test samples: moderate cognitive impairment (MCI), AD, and healthy controls (HC). The outcomes of three modes of classic classification methods, including KNN, SVM, Decision Tree, and LDA approaches, are documented, as well as the final feature employed in each. Finally, we show the CNN architecture for AD patient classification. Output assessment is used to show the results. Other techniques are outperformed by the given CNN and DT.

## 1. Introduction

The brain is the body's most important organ. The disorders that affect the brain are extremely important to manage since, in most situations, once alterations occur, they are irreversible in extreme circumstances. Dementia is defined as the loss of cognitive and functional thinking abilities. The most prevalent cause of dementia is AD. The AD strikes people in their mid-60s. Alzheimer's disease affects more than 5.5 million individuals worldwide [1]. Memory loss, language problems, and behavioral changes are all indications of AD. Symptoms of the nonmemory part include trouble locating words, eye problems, decreased cognition, and poor judgment. Brain imaging, cerebrospinal fluid, and blood are the biological signs. Normal age-related decrease in cognitive function, which is more gradual and associated with less impairment, should be distinguished from AD. The disease frequently begins with little symptoms and progresses to serious brain damage. Dementia affects people differently; therefore their abilities deteriorate at varying rates. Early and reliable identification of AD is advantageous to disease management. Neuroimaging techniques like magnetic resonance imaging (MRI) and computed tomography (CT), as well as single-photon emission computed tomography (SPECT) and positron emission tomography (PET), can be utilized to rule out other forms of dementia or subtypes. It has the potential to forecast the progression of prodromal into AD. Neurologists can use medical image processing and machine learning methods to see if a person is developing AD. Image segmentation and classification are critical tasks in MRI data analysis for detecting AD [2]. Structural MRI (SMRI) provides visual information regarding the atrophic areas of the brain caused by the tissue level abnormalities that underpin AD/MCI. PET measures cerebral glucose metabolism, which is a reflection of functional brain activity [3]. The quantity of amyloid beta-protein and amyloid tau tangles accumulated in the cerebrospinal fluid (CSF) is an early predictor of AD. SMRI has already been shown to be sensitive to presymptomatic illness and might be used as a disease biomarker [4]. MRI appears to be the most sensitive imaging examination of the brain in everyday clinical practice. It provides information on gray matter, white matter, and CSF morphology. Structural MRI can record atrophic brain areas noninvasively, allowing us to see anatomical alterations in the brain. As a result, they have been recognized as a possible indication of illness development, and ML approaches for disease detection are being researched extensively [5].

The MRI scan can be utilized in image processing to evaluate the likelihood of early detection of AD. Intensity adjustment, *K*-means clustering, and the region growing method are image processing techniques used in MRI to extract white and gray matter [6]. The same approach may be used to compute brain volume. Because the raw MRI brain image is too large to be utilized for classification, the MR images must be preprocessed before feature extraction and classification can be performed for illness diagnosis. Through the warping of labeled atlas, one of the most generally used approaches is to divide the image into numerous anatomical areas, that is, regions of interest (ROIs), and the regional measurements, such as volumes, are calculated as the features for AD classification [7]. To identify the most discriminative features from ROIs for multimodality classification of AD/MCI, a discriminative multitask algorithm was presented. In ML, each data item should be characterized as a feature vector.

There are numerous research advocated extracting various characteristics from MRI scans and then classifying the resulting vectors. The quality of the produced feature vectors is, nevertheless, reliant on image preprocessing due to registration errors and noise. As a result, domain knowledge is required to extract discriminative features. CNN's layered design has a big influence on its performance. Greater classification accuracy is anticipated to arise from a layer structure that is better suited for MRI images. The input images in this article are Two-Dimensional Discrete Wavelets (2D-DWT). The Time-Dependent Power Spectrum Descriptors (TD-PSD) model is used to represent the subbanded wavelet coefficients. The primary property vector is made up of the characteristics of the TD-PSD model. Based on classification algorithms, the collected characteristics are applied independently to present AD classifications. The classification is used to determine the kind of tumor. For feature extraction of wavelet subbands from three sets of mild cognitive impairment (MCI), AD, and HC test data, we employed the TD-PSD technique.

## 2. Literature Review

For diagnosing AD, feature vectors from MRI images must be extracted. Several feature extraction techniques have been proposed in the recent decade since the outcome of ML is determined by the extracted feature vectors. Employing many specified templates, Liu et al. [8] retrieved multiview feature representations for subjects and divided subjects within a particular class into distinct subclasses in each view space. Support vector machine-based (SVM) ensemble learning was used. Suk et al. proposed a multitask and multikernel SVM learning approach for a stacked autoencoder with a deep-learning-based feature representation [9]. Due to registration mistakes and noise, the quality of the recovered features is dependent on image preprocessing. As a result, domain knowledge is required while extracting discriminative features. It takes a long time and a lot of effort to acquire hand-crafted features. More crucially, hand-crafted features seldom generalize well. As a consequence, this study recommends employing deep learning to extract data characteristics. Sadeghipour and Sahragard [10] developed a novel approach for facial identification that is based on an enhanced SIFT algorithm. Acharya et al. [11] created an ML system that can detect AD symptoms in a brain scan. For classification, the system combined MRI with a variety of feature extraction techniques. The T2 imaging sequence was used to get the images. Filtering, feature extraction, Student's *t*-test-based feature selection, and *k*-Nearest Neighbor- (KNN-) based classification were among the quantitative approaches used in the paradigm. The findings revealed that when compared to other approaches, the Shearlet Transform (ST) feature extraction methodology provides better outcomes for Alzheimer's diagnosis. With the ST + KNN approach, the suggested tool achieved 94.54 percent accuracy, 88.33 percent precision, 96.30 percent sensitivity, and 93.64 percent specificity. According to Sadeghipour et al. [12], combining fireflies with intelligent systems would lead to breast cancer detection. The results show that by comparing the performance of the suggested system to other methods, it is evident that it is superior in both performance and accuracy. Sadeghipour and Moradisabzevar [13] investigated the development of intelligent toy cars as a method of screening children with autism. The results show that the screening of autistic children was 100 percent accurate. The study by Zhou et al. [14] investigated probabilistic inflection points for the decomposition of LiDAR hidden echo signals. Yan et al. [15] examined the structure and in vitro test results of waxy and regular maize starches after thermal processing using plasma-activated water. Eslami and Yun [16] have developed a novel approach called A + MCNN and have compared it to four commonly used deep classifiers in the transportation domain as well as the standard CNN classifier. Sadeghipour and Hatam [17] developed the XCSLA System to help in the diagnosis of diabetes. Hassantabar et al. [18] implemented three deep learning-based methods using X-ray images of the lungs to detect and diagnose COVID-19 patients. According to Sadeghipour and Hatam [19], the Expert Clinical System for Diagnosing Obstructive Sleep Apnea with Help from the XCSR Classifier helps diagnose this sleep disorder. Abadi et al. [20] have proposed a hybrid swarm algorithm and genetic algorithm (HSSAGA) model for solving nurses' scheduling and designation issues. In comparison with state-of-the-art approaches, this algorithm outperforms the suggested test function algorithm. Odusami et al. [21] suggested a deep-learning-based technique for predicting MCI, early MCI (EMCI), late MCI (LMCI), and AD. On the EMCI versus AD, LMCI versus AD, and MCI versus EMCI classification scenarios, the fine-tuned ResNet18 network achieved classification accuracy of 99.99%, 99.95%, and 99.95%, respectively, on the fine-tuned ResNet18 network. In terms of accuracy, sensitivity, and specificity, the suggested model outperformed other well-established models in the literature. Sharifi et al. [22] described a technique for diagnosing weary and exhausted feet using digital footprint photos. The current CNN technique outperforms existing methods and may be employed in the development of future fatigue detection systems. Furthermore, a conclusion neural network can be applied to the detection of tumors [23], the scheduling problems for health care systems [24], and the optimization of users based on a clustering method [25]. A new approach to penetration testing based on extended classifier networks has been proposed by Yazdani et al. [26]. A model of an application created for mobile Android systems was provided by Lauraitis et al. [27], which may be used to examine central nervous system movement problems occurring in individuals suffering from Huntington's, Alzheimer's, or Parkinson's illnesses. Specifically, the model detects tremors as well as cognitive deficits through the use of touch and visual stimulation modalities, among other things. According to the findings, the adoption of intelligent applications that may assist in the evaluation of neurodegenerative illnesses is a significant advancement in medical diagnostics and should be encouraged. According to Sadeghipour et al. [28], the xcsla system can be used to develop an intelligent diabetes diagnosis system. According to the results of the program implementation document (pid) on databases, the proposed technique can detect diabetes more accurately than the conventional xcs system, the Elman neural network, svm clustering, knn, c4.5, and ad tree. Farahanipad et al. [29] developed a pipeline for the identification of hand 2D keypoints using unpaired image-to-image translation. In Shi et al.'s [30] study, they investigated the effect of ultrasonic intensity on the structure and characteristics of sago starch complexes and their implications for the quality of Chinese steamed bread. Sadeghipour et al. [31] developed a new expert clinical method for the diagnosis of obstructive sleep apnea using the XCSR classifier. Rezaei et al. [32] used depth images to automate mild segmentation of hand parts. According to the results, a model without segmentation-based labels may achieve a mIoU of 42%. Quantitative and qualitative findings support our method's efficiency.

Yue et al. [33] use automated anatomical labeling (AAL) template to divide the brain into 90 regions of interest (ROIs). They choose the informative voxels in each ROI with a baseline of their values and arrange them into a vector to divide the uninformative data. The first stage characteristics were then chosen based on the voxel correlation between distinct groups. The fetched voxels were then put into a convolutional neural network (CNN) to understand the profoundly hidden properties of each subject's brain features maps. The testing findings showed that the suggested technique was reliable and had a promising performance when compared to other methods in the literature.

For increasing classification accuracy and identifying high-order features that potentially provide pathological information, Li et al. [44] used a novel feature extraction approach known as radiomics. As a consequence, they defined ROIs as brain regions mostly dispersed in the temporal, occipital, and frontal areas. A total of 168 radiomic characteristics of Alzheimer's disease were found to be stable (alpha >0.8). The maximum accuracies for categorizing AD versus HC, MCI versus HCs, and AD versus MCI were 91.5 percent, 83.1 percent, and 85.9 percent, respectively, in the classification trial. Silva et al. [46] suggested a model for diagnosing AD based on deep feature extraction for MRI classification. The goal of this model was to distinguish between AD and HC. For extracting the best characteristics of the selected region, the CNN architecture was also developed in three convolutional layers. The model's effectiveness and reliability for the diagnosis of AD were shown by a comparison study with previous studies in the literature.  Table 1 lists several more techniques.

## 3. Methods and Materials

### 3.1. Discrete Wavelet Transform (DWT)

If *f*(*x*) ∈ *L*^2^(*R*)  is a wavelet expansion function that is connected to wavelet *ψ*(*x*) and scaling *φ*(*x*), we get [47]

(1) $$\begin{array}{c} f\left(x\right)=\sum_{k}c_{j_{0}}\left(k\right)\varphi_{j_{0},k}\left(x\right)+\sum_{j=j_{0}}^{\infty }\sum_{k}d_{j}\left(k\right)\psi_{j,k}\left(x\right). \end{array}$$

**Figure 1:**
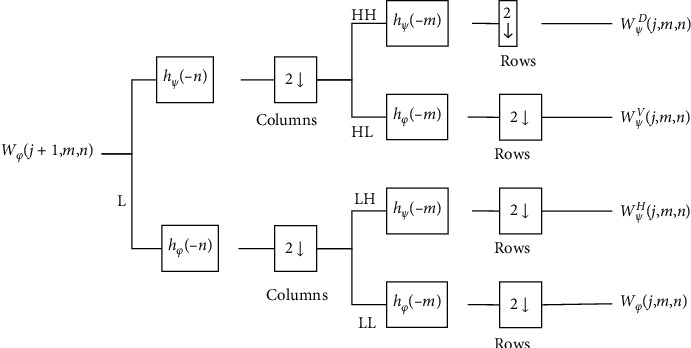
The two-dimensional DWT diagram.

 *c*_*j*_0__(*k*)'s are scaling coefficients, and *j*_0_ is a starting counter. The *d*_*j*_(*k*) coefficients are wavelet coefficients (see Figure 1). The following are the expansion coefficients:

(2) $$\begin{array}{c} c_{j_{0}}\left(k\right)=\langle f|\left(x\right)|,|{\tilde{\varphi }}_{j0,k}|\left(x\right)\rangle =\int f\left(x\right){\tilde{\varphi }}_{j0,k}\left(x\right)\text{d}x, \end{array}$$

(3) $$\begin{array}{c} d_{j}\left(k\right)=\langle f|\left(x\right)|,|{\tilde{\psi }}_{j,k}|\left(x\right)\rangle =\int f\left(x\right){\tilde{\psi }}_{j,k}\left(x\right)\text{d}x, \end{array}$$

(4) $$\begin{array}{c} {\tilde{\varphi }}_{j,k}\left(x\right)=2^{j/2}\tilde{\varphi }\left(2^{j}n-k\right), \end{array}$$

It is also known as the discrete wavelet transform of *f*(*x*)  if the expansion function is a series of crisp numbers. The expansion series is represented by equations (2) and (3) (DWT pair) [47, 48]:

(5) $$\begin{array}{c} W_{\varphi }\left(j_{0},k\right)=\frac{1}{\sqrt{M}}\sum_{x=0}^{M-1}f\left(x\right){\tilde{\varphi }}_{j_{0},k}\left(x\right),\\ W_{\psi }\left(j,k\right)=\frac{1}{\sqrt{M}}\sum_{x=0}^{M-1}f\left(x\right){\tilde{\psi }}_{j,k}\left(x\right)\;j\geq j_{0},\\ f\left(x\right)=\frac{1}{\sqrt{M}}\sum_{k}W_{\varphi }\left(j_{0},k\right)\varphi_{j_{0},k}\left(x\right)+\frac{1}{\sqrt{M}}\sum_{j=j_{0}}^{\infty }\sum_{k}W_{\psi }\left(j,k\right)\psi_{j,k}\left(x\right), \end{array}$$

where  *f*(*x*), *φ*_*j*_0_,*k*_(*x*), and *ψ*_*j*,*k*_(*x*) are discrete variables, *x* = 0, 1,…, *M* − 1*, j* = 0, 1,…, *J* − 1, *k* = 0, 1, 2,…, *M* − 1 functions, where *M* is the number of samples to be converted, and *J* is the number of transformation levels; it equals  2^*J*^. To construct a 1D scaling function *ϕ* and associated wavelet *ψ* [39], 2D, *φ*(*x*, *y*), and 3D, *ψ*^*H*^(*x*, *y*), *ψ*^*V*^(*x*, *y*), and *ψ*  ^*D*^(*x*, *y*), are usually necessary.

(6) $$\begin{array}{c} \varphi \left(x,y\right)=\varphi \left(x\right)\varphi \left(y\right),\\ \psi^{H}\left(x,y\right)=\psi \left(x\right)\varphi \left(y\right),\\ \psi^{V}\left(x,y\right)=\varphi \left(y\right)\psi \left(x\right),\\ {\psi \;}^{D}\left(x,y\right)=\psi \left(x\right)\psi \left(y\right). \end{array}$$

A two-level wavelet transformation creates four subbands, as seen in Figure 1. In this diagram 2↓,  *ψ*^*H*^, *ψ*^*V*^, and *ψ*  ^*D*^ indicated deviations along horizontal, vertical, and diagonal boundaries, respectively. Digital filtration and downsamplers can be used to perform 2D-DWT. The additional subbands are produced using discrete 2D scaling functions and 1D-FWT on *f* (*x*, *y*) [49].

### 3.2. Feature Extraction

The discrete Fourier transform (DFT) is supposed to explain the signal trace as a function of frequency *X*[*k*] as a product of the sampled representation of the signal as *x*[*j*] with *j*=1,2,…*N*, length *N*, and sampling frequency fs Hz. If we remember Parseval's theorem, the sum of the square of the function equals the whole square of its transformation. We begin the feature extraction procedure.

(7) $$\begin{array}{c} \sum_{j=0}^{N-1}{\left|x\left[j\right]\right|}^{2}=\frac{1}{N}\sum_{k=0}^{N-1}\left|X\left[k\right]X^{*}\left[k\right]\right|=\sum_{k=0}^{N-1}P\left[k\right], \end{array}$$

*P*[*k*] is the phase-excluded power spectrum, according to the preceding equation. This implies that multiplying *X*[*k*] by the *X*^*∗*^[*k*] conjugate divided by *N* yields the frequency index.

*P*[*k*] is the phase-excluded power spectrograph; that is, *X*[*k*] has its conjugate  *X*^*∗*^[*k*], which is separated by *N*, which is compounded by *k*, and frequency index. The Fourier transform's whole notion of frequency is usually thought to be symmetrical with respect to zero frequency. It has similar sections that cover both positive and negative frequencies. This symmetry does not apply to all frequencies in the spectrum, including positive and negative ones. Because we do not have comprehensive accessibility to the time domain, we cannot employ spectral power from there. By the statistical approach of the frequency distribution model, all irregular moments are also zero, corresponding to the concept of a one-minute *m* of the power spectral density of order *n P*[*k*].

(8) $$\begin{array}{c} m_{n}=\sum_{k=0}^{N-1}k^{n}P\left[k\right]. \end{array}$$

The Parseval theorem might indeed be used when *n* = 0 is used. For nonzero values of *n*, the Fourier transform time-differentiation feature could be applied. The *n*^'th^ means multiplying the *k* by the spectrum to the *n*^'th^ power, according to this feature. The derivative of a time-domain function is alluded to as Δ^*n*^ for distinct time signals.

(9) $$\begin{array}{c} F\left[\Delta^{n}x\left[j\right]\right]=k^{n}X\left[k\right]. \end{array}$$

*Root Squared Zero-Order Moment* ($\bar{m_{0}}$). This is a function that displays the frequency domain's total power and looks like this
  (10) $$\begin{array}{c} \bar{m_{0}}=\sqrt{\sum_{j=0}^{N-1}x{\left[j\right]}^{2}s}. \end{array}$$
All channels could standardize their related zero-order moments by splitting them into zero-order moments.
*Root Squared Second- and Fourth-Order Moments*. The second time is utilized as power, but it is subsequently shifted to  *k*^2^*P*[*k*], which refers to the frequency function:
  (11) $$\begin{array}{c} \bar{m_{2}}=\;\sqrt{\sum_{k=0}^{N-1}k^{2}P\left[k\right]}=\sqrt{\frac{1}{N}\sum_{k=0}^{N-1}{\left(kX\left[k\right]\right)}^{2}}=\sqrt{\sum_{j=0}^{N-1}{\left(\Delta x\left[j\right]\right)}^{2}}. \end{array}$$
The moment is obtained by repeating this approach:
  (12) $$\begin{array}{c} \bar{m_{4}}=\;\sqrt{\sum_{k=0}^{N-1}k^{4}P\left[k\right]}=\sqrt{\sum_{k=0}^{N-1}{\left(\Delta^{2}x\left[j\right]\right)}^{2}}. \end{array}$$
The overall energy of the signal is reduced when the second and fourth signals are taken into account. For decreasing the noise impact on all moment-based features, to normalize the domains of *m*_0_,  *m*_2_,   and *m*_4_, we perform the following power transformation:
  (13) $$\begin{array}{c} m_{0}=\frac{{\bar{m_{0}}}^{\lambda }}{\lambda },\\ m_{2}=\frac{{\bar{m_{2}}}^{\lambda }}{\lambda },\\ m_{4}=\frac{{\bar{m_{4}}}^{\lambda }}{\lambda }. \end{array}$$
The experimental value of *λ* is set to 0. As a result of these settings, the first three features extracted are as follows:
  (14) $$\begin{array}{c} f_{1}=\log\left(m_{0}\right),\\ f_{2}=\log\left(m_{0}-m_{2}\right),\\ f_{3}=\log\left(m_{0}-m_{4}\right). \end{array}$$
*Sparseness*. This feature calculates the quantity of vector energy in a small number of additional components. It is then followed by
  (15) $$\begin{array}{c} f_{4}=\log\left(\frac{m_{0}}{\sqrt{m_{0}-m_{2}}\sqrt{m_{0}-m_{4}}}\right). \end{array}$$
A feature shows a vector with all elements equivalent to a zero-sparseness index, i.e.,  *m*_2_, and *m*_4_=0, due to differentiation and  log(*m*_0_/*m*_0_)=0. All other sparseness levels, on the other hand, should have a value greater than zero.
*Irregularity Factor (IF)*. The ratio of peak numbers divided by zero-crossings up is expressed by this metric. A random signal's number of upward zero-crossings (ZC) and number of peaks (NP) can only be characterized in terms of spectral instances. The following is how the appropriate feature should be written:
  (16) $$\begin{array}{c} f_{5}=\frac{\text{ZC}}{\text{NP}}=\frac{\sqrt{m_{2}/m_{0}}}{\sqrt{m_{4}/m_{2}}}=\sqrt{\frac{m_{2}^{2}}{m_{0}m_{4}}}=\frac{m_{2}}{\sqrt{m_{0}m_{4}}}. \end{array}$$
*Covariance (COV)*. Our COV function is described as the standard deviation on arithmetic averages divided by the standard deviation on arithmetic averages:
  (17) $$\begin{array}{c} f_{6}=\log\left(\frac{\sqrt{\sum_{j=0}^{N-1}{\left(x-\bar{x}\right)}^{2}/n}}{\bar{x}}\right). \end{array}$$
*Teager energy operator (TEO)*. It mainly depicts the signal amplitude and instantaneous fluctuations, which are particularly sensitive to even little variations. TEO has been proposed as a method for modeling nonlinear speech signals. It was later widely employed in the audio signal processing industry. It is made up of the following parts:
  (18) $$\begin{array}{c} f_{7}=\log\left(\Psi \left(x\left[j\right]\right)\right)\;=\log\left(\overset{N-1}{\underset{j=0}{\sum }}x^{2}\left[j\right]-x\left[j-1\right]x\left[j+1\right]\right). \end{array}$$

### 3.3. Proposed Feature Extraction Methods

**Figure 2:**
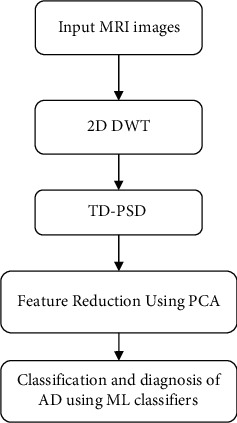
The block diagram of the proposed method.

The goal of this research is to apply machine learning algorithms to identify Alzheimer's disease.  Figure 2 show the block diagram of the proposed method. To begin, we employed a two-stage 2D-DWT to break down input images into wavelet subbands. The obtained wavelet coefficients are utilized to derive classification features. The TD-PSD model is then used to extract features, with the first step using HH1, HL1, LH1, and the second stage using LL2, HH2, HL2, and LH2. The PCA approach is employed to diminish the number of features, and then AD is categorized using multiple machine learning algorithms using the retrieved feature. The following is the pseudocode for the provided method (Algorithm 1).

## 4. Results

### 4.1. Data Collection

In AD, structural MR imaging results demonstrated microscopic neurodegeneration and are a measure of brain atrophy (loss of synapses, dendritic processes, and neurons). In volumetric or voxel-based assessments of brain atrophy, the degree of atrophy and the extent of cognitive impairment are closely associated. There is a relationship between cognitive decline and brain atrophy. Atrophy does not appear to be exclusive to AD on MR images. The degree of hippocampal atrophy, on the other hand, is highly correlated with autopsy Braak staging [50]. Braak staging of neurofibrillary tangles in antemortem MR imaging and postmortem AD staging match to the topographic distribution of atrophy on MR images (medial, basal, and lateral temporal lobes, as well as the medial parietal cortex) [51]. The data collection includes atrophy and clinical stages of AD. There is negligible atrophy in the cognitively normal control individual, while there is significant atrophy in the AD patient. The MCI individual, on the other hand, has an intermediate amount of atrophy. On Kaggle [52], the dataset is accessible online. The MRI images are 256 × 256 PNG grayscale images that have been utilized to analyze and evaluate AD in three classes: AD, MCI, and an HC group.

### 4.2. Feature Extraction and Reduction

**Figure 3:**
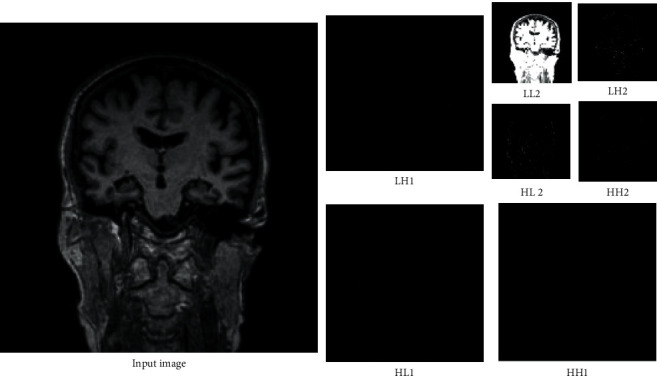
The subbands of the discrete wavelet transformation for an input image. HH: high pass-high pass subband, HL: high pass-low pass subband, LH: low pass-high pass, LL: low pass-low pass, 1: first level, and 2: second level.

In this section, the process of feature extraction is described. Based on the conceptual diagram of Figure 2 and pseudocode, the first step in the presented method is wavelet decomposition. The results of decomposition are presented in Figure 3. Regarding Figure 3, a two-level decomposition is done for each input image. From the first step, three subbands of low-high (LH1), high-low (HL1), high-high (HH1), and from the second step low-low (LL2), LH2, HL2, and HH2 are used for feature extraction.

**Figure 4:**
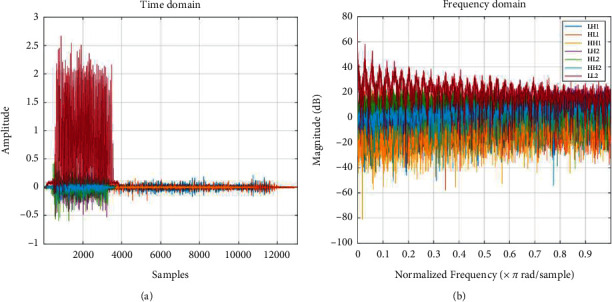
The frequency (a) and the amplitude (b) of the subbands for DWT for and input image.

In the next step, each subband matrix is reshaped to a vector, and all the zeros are removed from the vectors. The final vectors are our pseudotime series that are used for feature extraction. The properties of the seven subbands are presented in Figure 4. Based on the amplitude and frequency of subbands, the LL2 subbands include the maximum number of points and properties of input images. However, all subbands are consequential in this diagnosis.

**Figure 5:**
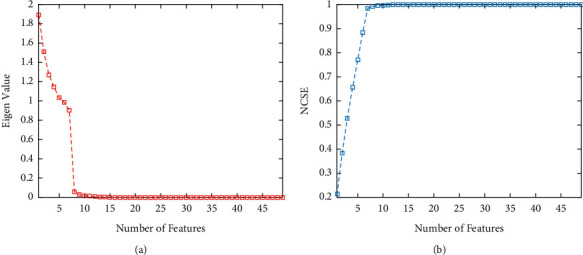
Results of feature reduction based on PCA method. (a) Eigen value of the features. (b) Normalized cumulative summation of Eigen values.

Based on the feature extraction results, each image has 49 features (7 subbands with 7 TD-PSD features). Moreover, principal component analysis is employed to reduce the features. Based on Figure 5, the first seven features include almost 100% effect of all features. Consequently, the number of features is reduced to 7 based on the screen plot in Figure 5(a). Moreover, the cumulative value of the eigenvalue is presented in Figure 5(b).

### 4.3. Results of Classification

**Figure 6:**
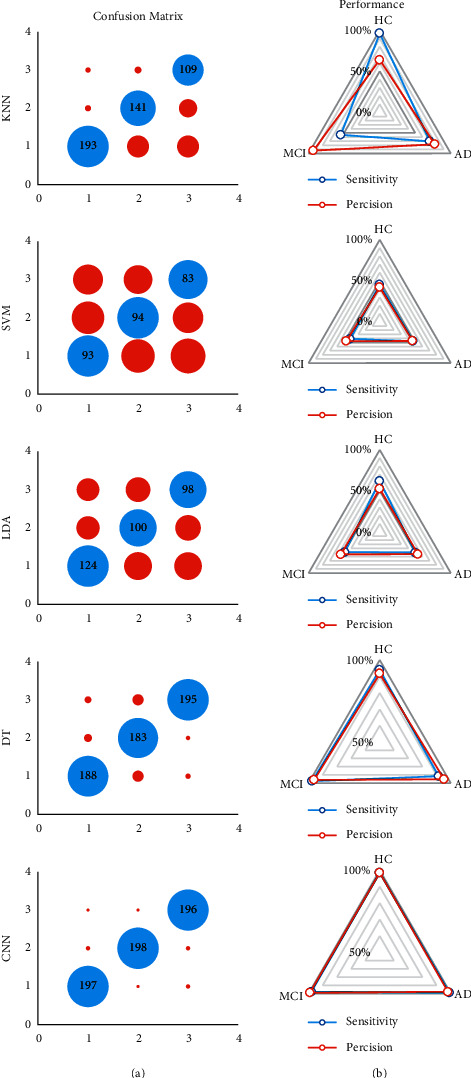
The confusion matrixes (a) and the performance plots (b) of the machine learning methods based on the WTD-PSD. The labels 1, 2, and 3 display the HC, AD, and MCI.

In this section, the classification is done using different machine learning methods. The input layer of the classification methods is seven reduced features of the images, and the output layer is the three-class label of AD, MCI, and HC. Total 600 MRI images are employed for the classification of AD. The confusion matrixes of the presented methods are illustrated in Figure 6. The blue balls show the true values, and the red balls are the false value of the classification. Moreover, labels 1, 2, and 3 display the HC, AD, and MCI, respectively. Regarding the results of the KNN method, from 200 input HC, AD, and MCI images 193, 141, and 109 are diagnosed correctly. Based on the results, the sensitivity of the KNN for diagnosing Alzheimer's disease for HC is acceptable. Depending on the results, the SVM and LDA approaches reached the weak result for the diagnosis of AD. However, the results of DT show that the sensitivity of the method is 94%, 91.5%, and 97.5%, respectively. It means that the WTD-PSD is compatible with the DT approach for this problem. In other words, 188, 183, and 195 MRI images from HC, AD, and MCI are detected, respectively. Moreover, the precision of the method is 91.70%, 95.30%, and 96.10% for HC, AD, and MCI, accordingly.

**Figure 7:**
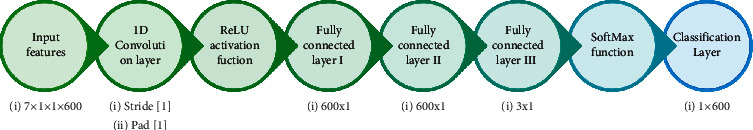
The CNN architecture for classification Alzheimer disease based on the WTD-PSD.

To approve the presented feature, we used CNN architecture also for this problem. The architecture of the CNN is presented in Figure 7.

Input layer includes

- Seven reduced features of MCI, AD, and HC
- Input matrix 4D [ 7 × 1 × 1×600]

The hidden layers include

- 1D convolution layer
- Rectified linear unit layer
- Fully connected layer (600)
- Fully connected layer (600)
- Fully connected Layer (3)
- Output layers include
- SoftMax layer
- Classification layer 1D [1 × 600]

**Figure 8:**
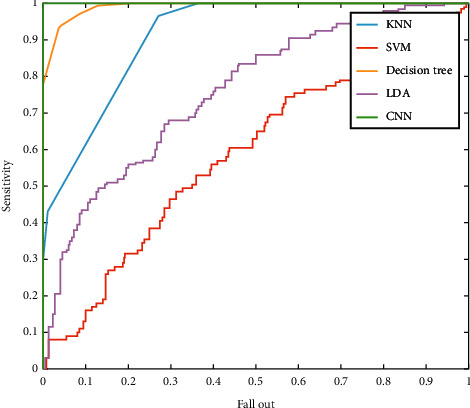
The ROC curve for the classification based on the WTD-PSD and machine learning classifiers.

**Figure 9:**
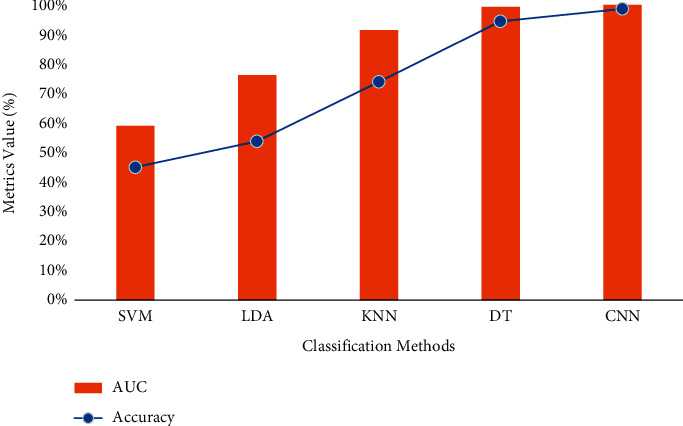
The accuracy and AUC value for the presented machine learning methods.

Based on the results of the CNN classifier, the sensitivity of the method is 94%, 91.5%, and 97.5% for HC, AD, and MCI, respectively. Moreover, from 200 images for each class, 197, 198, and 196 are detected accordingly. Finally, the precision is 91.7%, 95.3%, and 96.1%, with the same respect. To compare the presented machine learning method for diagnosing Alzheimer's disease, the ROC is depicted in Figure 8. The horizontal axis of the ROC curve represents the rate of the false-positive index depending on the HC class. The genuine positive rate is shown by the vertical axis. The best classifier has the highest rate of true positives and the lowest number of false positives. Based on the results, the CNN and DT method shows the two best classifiers for the presented features. Moreover, the area under the curve (AUC) value is an index to compare the classifiers. The AUC and the accuracy of the machine learning classifiers are presented in Figure 9. Centered on the results, the accuracy of SVM, LDA, KNN, DT, and CNN is 45%, 53.70%, 73.80%, 94.33%, and 98.50%, respectively. Based on this chart, the CNN architecture with the highest accuracy and AUC is the more accurate and compatible method for diagnosing Alzheimer's disease using the WTD-PSD. Moreover, DT is the second method with a higher AUC.

## 5. Discussion

Since each data sample in ML should be defined as a feature vector, several researches have recommended extracting various features from MRI scans and then categorizing the vectors generated as a consequence of this process. Image preprocessing, on the other hand, is necessary to increase the quality of the recovered feature vectors because of registration mistakes and noise in the image. It is necessary to have domain knowledge in order to derive discriminative qualities. Discrete wavelet is employed as the input image in this study, and it has a two-dimensional representation. The subbanded wavelet coefficients are modeled using the Time-Dependent Power Spectrum Descriptors model, which is implemented in MATLAB. Each of the attributes of the TD-PSD model is represented by one of the leading property vectors. The collected characteristics are utilized in an autonomous manner to construct AD classifications, which are based on classification algorithms. On the basis of the findings, the accuracy of SVM, LDA, KNN, DT, and CNN are correspondingly 45 percent, 53.70 percent, 73.80 percent, 94.33 percent, and 98.50 percent. SVM is the most accurate of the five models. According to this figure, the CNN architecture with the highest accuracy and AUC is the most accurate and compatible technique for diagnosing Alzheimer's disease when utilizing the WTD-PSD than the other two methods. Furthermore, DT is the second most accurate approach with a larger AUC.

## 6. Conclusion

Many studies have advised extracting numerous features from MRI scans and then categorizing the resulting vectors since each data sample in ML should be described as a feature vector. However, image preprocessing is required to improve the quality of the recovered feature vectors due to registration errors and noise. For extracting discriminative characteristics, domain knowledge is required. The Two-Dimensional Discrete Wavelet is used as the input image in this work. The Time-Dependent Power Spectrum Descriptors model is used to model the subbanded wavelet coefficients. The leading property vector is made up of the characteristics of the TD-PSD model. Based on classification algorithms, the extracted features are applied independently to present AD classifications. The classification is used to determine the kind of tumor. We extracted wavelet subband features from three sets of MCI, AD, and HC data using the TD-PSD method. According to the KNN approach, images 193, 141, and 109 are correctly detected from 200 input HC, AD, and MCI images. According to the findings, the KNN's sensitivity for identifying AD in HC patients is adequate. According to the findings, the SVM and LDA approaches yielded a poor outcome for diagnosing AD. The DT findings, on the other hand, demonstrate that the method's sensitivity is 94 percent, 91.5 percent, and 97.5 percent, respectively. It indicates that for this issue, the WTD-PSD is compatible with the DT technique. In other words, MRI images from HC, AD, and MCI are observed in 188, 183, and 195, respectively. Furthermore, the method's precision for HC, AD, and MCI is 91.70 percent, 95.30 percent, and 96.10 percent, respectively. According to the CNN classifier's findings, the method's sensitivity for HC, AD, and MCI is 94 percent, 91.5 percent, and 97.5 percent, respectively. Furthermore, out of 200 images, 197, 198, and 196 are recognized for each class. Eventually, 91.7 percent, 95.3 percent, and 96.1 percent precision are achieved. The CNN architecture with the greatest accuracy and AUC is the more accurate and compatible technique for diagnosing AD utilizing the WTD-PSD, according to this figure. DT is also the second approach with the highest AUC.

## Figures and Tables

**Algorithm 1:**
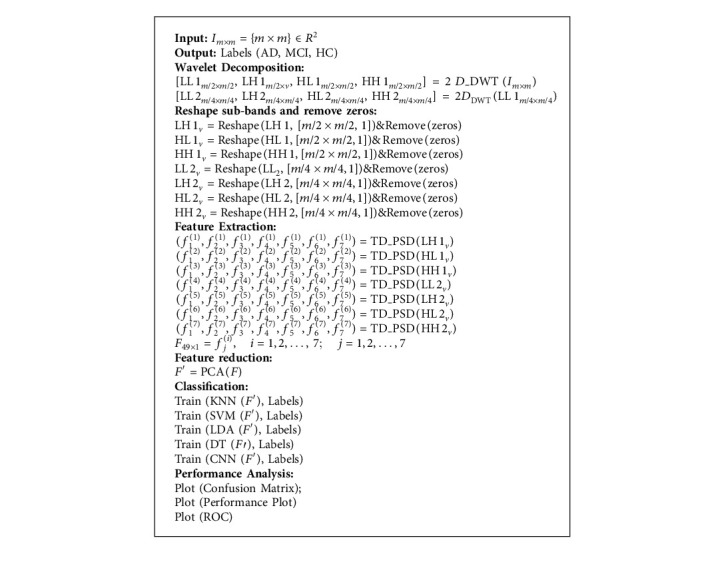
The pseudocode for the proposed method.

**Table 1:** Summary of method for diagnosis of Alzheimer using computer-aided approach.

Author	Year	Data	Classes	Feature extraction	Classifier
Yang et al. [34]	2021	Magnetoencephalography	AD, MCI, HC	Space–frequency–time domain feature extraction	3-NN and QBNC
Hedayati et al. [35]	2021	3D-MRI	AD, MCI, HC	Ensemble of pre-trained auto encoder	CNN
Biagetti et al. [36]	2021	EEG signal	AD, HC	Robust-PCA	KNN, DT, SVM, NB
Chen and Xia [37]	2021	sMRI	AD, MCI, HC	Deep feature extraction	CNN
Ahmadi et al. [38]	2021	MRI	Low, mild, moderate, severe stage	Brain tumor diagnosis	Fuzzy logic, CNN
Amini et al. [39]	2021	fMRI	Low, mild, moderate, severe stage	Robust multitask feature extraction method	KNN, SVM, DT, LDA, CNN
Janghel and Rathore [40]	2020	fMRI, PET	AD, HC	Image map	SVM, DT, LDA, CNN
Ahmadi et al. [41]		MRI	Low, mild, moderate, severe stage	Tumor area segmentation	QAIS-DSNN
Parmar et al. [42]	2020	fMRI	AD, EMCI, LMCI, HC	Spatiotemporal feature extraction	3D-CNN
Ahmadi et al. [43]	2021	MRI	Low, mild, moderate, severe stage	Brain tumor diagnosis	CNN
Li et al. [44]	2019	18F-FDG PET imaging	AD, MCI, HC	High-order radiomic features extraction	SVM
Yue et al. [33]	2019	MRI	AD, MCI, HC	Voxel-based hierarchical method	CNN
Acharya et al. [11]	2019	MRI	AD, HC	Shearlet transform, curvelet, contourlet, complex wavelet, discrete wavelet, empirical wavelet, dual tree complex wavelet	KNN
Fiscon et al. [45]	2018	EEG signal	AD, MCI, HC	Fast Fourier transform, discrete wavelet transform	DT

## Data Availability

Data are available and can be provided upon direct request to the corresponding author at ali.taghavi.eng@iauctb.ac.ir.
